# Personalized early detection and prevention of breast cancer: ENVISION consensus statement

**DOI:** 10.1038/s41571-020-0388-9

**Published:** 2020-06-18

**Authors:** Nora Pashayan, Antonis C. Antoniou, Urska Ivanus, Laura J. Esserman, Douglas F. Easton, David French, Gaby Sroczynski, Per Hall, Jack Cuzick, D. Gareth Evans, Jacques Simard, Montserrat Garcia-Closas, Rita Schmutzler, Odette Wegwarth, Paul Pharoah, Sowmiya Moorthie, Sandrine De Montgolfier, Camille Baron, Zdenko Herceg, Clare Turnbull, Corinne Balleyguier, Paolo Giorgi Rossi, Jelle Wesseling, David Ritchie, Marc Tischkowitz, Mireille Broeders, Dan Reisel, Andres Metspalu, Thomas Callender, Harry de Koning, Peter Devilee, Suzette Delaloge, Marjanka K. Schmidt, Martin Widschwendter

**Affiliations:** 1 Department of Applied Health Research, Institute of Epidemiology and Healthcare, University College London, London, UK; 2 Department of Public Health and Primary Care, University of Cambridge, Cambridge, UK; 3 Epidemiology and Cancer Registry, Institute of Oncology Ljubljana, Ljubljana, Slovenia; 4 Carol Franc Buck Breast Care Center, University of California, San Francisco, CA USA; 5 Division of Psychology & Mental Health, School of Health Sciences, University of Manchester, Manchester, UK; 6 Department of Public Health, Health Services Research and Health Technology Assessment, Institute of Public Health, Medical Decision Making and Health Technology Assessment, UMIT-University for Health Sciences, Medical Informatics and Technology, Hall in Tirol, Austria; 7 Division of Health Technology Assessment, Oncotyrol — Center for Personalized Cancer Medicine, Innsbruck, Austria; 8 Department of Medical Epidemiology and Biostatistics, Karolinska Institutet, Stockholm, Sweden; 9 Department of Oncology, Södersjukhuset, Stockholm, Sweden; 10 Wolfson Institute of Preventive Medicine, Barts and The London, Centre for Cancer Prevention, Queen Mary University of London, London, UK; 11 Division of Evolution and Genomic Sciences, University of Manchester, Manchester, UK; 12 Genomics Center, CHU de Québec — Université Laval Research Center, Québec, Canada; 13 Division of Cancer Epidemiology and Genetics, National Cancer Institute, Bethesda, MD USA; 14 Center of Family Breast and Ovarian Cancer, University Hospital Cologne, Cologne, Germany; 15 Max Planck Institute for Human Development, Center for Adaptive Rationality, Harding Center for Risk Literacy, Berlin, Germany; 16 Department of Oncology, University of Cambridge, Cambridge, UK; 17 PHG Foundation, Cambridge, UK; 18 IRIS Institute for Interdisciplinary Research on Social Issues, Paris, France; 19 Unicancer, Paris, France; 20 Epigenetic Group, International Agency for Research on Cancer (IARC), WHO, Lyon, France; 21 Division of Genetics and Epidemiology, Institute of Cancer Research, London, UK; 22 Department Medical Imaging, Institut Gustave Roussy, Villejuif, France; 23 Epidemiology Unit, Azienda USL di Reggio Emilia — IRCCS, Reggio Emilia, Italy; 24 Division of Molecular Pathology, Netherlands Cancer Institute, Antoni van Leeuwenhoek Hospital, Amsterdam, Netherlands; 25 Faculty of Medicine and Health Sciences, University of Antwerp, Antwerp, Belgium; 26 Department of Medical Genetics, National Institute for Health Research Cambridge Biomedical Research Centre, University of Cambridge, Cambridge, UK; 27 Department for Health Evidence, Radboud University Medical Center, Nijmegen, Netherlands; 28 Department of Women’s Cancer, Institute for Women’s Health, University College London, London, UK; 29 The Estonian Genome Center, Institute of Genomics, University of Tartu, Tartu, Estonia; 30 Department of Public Health, Erasmus MC, Rotterdam, Netherlands; 31 Department of Human Genetics, Department of Pathology, Leiden University Medical Centre, Leiden, Netherlands; 32 Breast Cancer Department, Gustave Roussy Institute, Paris, France; 33 Universität Innsbruck, Innsbruck, Austria; 34 European Translational Oncology Prevention and Screening (EUTOPS) Institute, Hall in Tirol, Austria

**Keywords:** Breast cancer, Cancer prevention, Cancer screening

## Abstract

The European Collaborative on Personalized Early Detection and Prevention of Breast Cancer (ENVISION) brings together several international research consortia working on different aspects of the personalized early detection and prevention of breast cancer. In a consensus conference held in 2019, the members of this network identified research areas requiring development to enable evidence-based personalized interventions that might improve the benefits and reduce the harms of existing breast cancer screening and prevention programmes. The priority areas identified were: 1) breast cancer subtype-specific risk assessment tools applicable to women of all ancestries; 2) intermediate surrogate markers of response to preventive measures; 3) novel non-surgical preventive measures to reduce the incidence of breast cancer of poor prognosis; and 4) hybrid effectiveness–implementation research combined with modelling studies to evaluate the long-term population outcomes of risk-based early detection strategies. The implementation of such programmes would require health-care systems to be open to learning and adapting, the engagement of a diverse range of stakeholders and tailoring to societal norms and values, while also addressing the ethical and legal issues. In this Consensus Statement, we discuss the current state of breast cancer risk prediction, risk-stratified prevention and early detection strategies, and their implementation. Throughout, we highlight priorities for advancing each of these areas.

## Abstract (web summary)

Risk-adapted approaches to breast cancer prevention and screening could potentially be more effective than universal approaches, which have important limitations. In this Consensus Statement, representatives of the European Collaborative on Personalized Early Detection and Prevention of Breast Cancer (ENVISION) discuss the current state of breast cancer risk prediction, risk-stratified prevention and early detection strategies, and their implementation. They also present the ENVISION recommendations on priorities for future research in each of these areas with the aim of stimulating and guiding risk-adapted breast cancer prevention and screening programmes.

## Introduction

Worldwide, breast cancer is the second most commonly diagnosed cancer, with approximately 2.1 million new diagnoses and almost 627,000 breast cancer-related deaths estimated to have occurred in 2018 (ref.^1^). Breast cancer is a biologically and clinically heterogeneous disease, with several recognized histotypes and molecular subtypes that have different aetiologies, profiles of risk factors, responses to treatments and prognoses^2–8^. In high-income countries, approximately 75% of breast cancers are diagnosed in postmenopausal women, although around 5–7% are diagnosed in women younger than 40 years of age^9,10^.

The risk of developing breast cancer varies among women. Genetic susceptibility, factors affecting levels of endogenous hormones (early age at menarche, later age at menopause, nulliparity, late age at first birth, having fewer children and shorter durations of breastfeeding), exogenous hormone intake (hormonal contraceptive use and hormone replacement therapy), lifestyle patterns (high alcohol intake, smoking and physical inactivity), anthropometric characteristics (greater weight, weight gain during adulthood and higher central body fat distribution), a high mammographic breast density and benign breast diseases (non-proliferative disease, proliferative disease without atypia and atypical hyperplasia) are all associated with an increased risk of breast cancer^11–14^. At an individual level, the mechanisms and relative contributions of these different risk factors to the development of breast cancer and also to particular subtypes of the disease are increasingly understood^15^.

Women with pathogenic germline mutations in cancer susceptibility genes — that is, in *BRCA1* or *BRCA2* (*BRCA1/2*) — may opt to undergo prophylactic bilateral mastectomy; primary chemoprophylaxis with tamoxifen or other selective oestrogen receptor modulators has also been recommended in this group, albeit the uptake is low^16^. Historically, members of this high-risk group have been identified on an opportunistic basis following self-referral of women with a family history of breast or ovarian cancer, or on the basis of an ancestry associated with an increased prevalence of clinically significant pathogenic variants of *BRCA1/2* (for example, in those of Jewish descent)^16^. Currently, genetic testing remains somewhat restricted for women with breast cancer; those with triple-negative, bilateral or young-onset disease might be offered a test at diagnosis, but most will be offered testing only if they also have a noted family history of the disease^16^. The 2019 US Preventive Services Task Force recommendations expand the population in which eligibility for genetic testing should be assessed to include women with a personal or family history of breast, ovarian, tubal or peritoneal cancer, in addition to women who have an ancestry associated with pathogenic *BRCA1/2* variants^17^.

At present, the mammographic screening programmes used for early detection of breast cancer in most high-income countries are based on the results of trials conducted at least 20–30 years ago^18–22^ and have age as the only entry criterion, although the starting and stopping ages (varying from 40 to 74 years) and the frequency of screens (yearly to triennially) differ between countries. This ‘one-size-fits-all’ approach does not take into account the heterogeneity of the breast cancer subtypes and of the risk in the population. Three decades of mammographic early detection have witnessed an increase in the incidence of early stage cancers with a low-risk tumour biology and an increase in the detection of in situ disease, without a concomitant proportionate decrease in incidence of advanced-stage disease^23,24^. Increasingly, calls have been made for a new approach to early detection with a focus on the identification of more consequential cancers and on avoiding the detection of indolent or ultra-low-risk disease^24,25^.

**Fig. 1:**
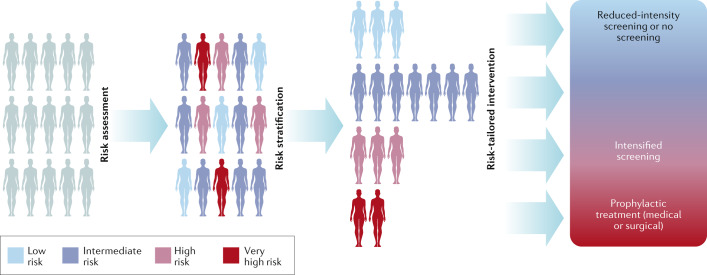
A schematic outlining a personalized approach to early detection and prevention of breast cancer. Women entering a personalized early detection programme would initially be assessed using a validated tool to determine their estimated risk of breast cancer. Subsequently, the women would be stratified into appropriate risk groups such that they can receive tailored interventions. This approach might mean that some women start mammographic screening at a younger age, have different screening intervals or have supplemental screening with another imaging modality, such as MRI. Women deemed to be at higher risk of breast cancer could, in addition, be offered prophylactic treatment. A healthy lifestyle would be recommended to all women, independent of risk level.

Personalized approaches to the prevention or early detection of breast cancer have emerged as highly promising strategies^26,27^. These programmes require risk assessment of each woman in the population, stratification of the population into several risk groups, assignment of the individuals to a specific risk group and tailoring of prevention and early detection interventions to each risk group^28^ (Fig. 1). Several international research consortia (Table 1) are studying ways to better understand, estimate and reduce breast cancer risk^29–32^, to use risk-based stratification to prevent consequential cancers^33,34^, to evaluate the benefit–harm trade-offs of such strategies^35^ and to assess the acceptability and feasibility of implementing risk-stratified prevention and early detection programmes^36–38^.

**Table 1:** Consortia participating in the ENVISION network and endorsing the recommendations herein

Acronym	Consortium	Description and/or aims of the consortium	Funder	Ref.
B-CAST	Breast Cancer Stratification	Define the influence of risk factors, including reproductive history, lifestyle, mammographic breast density and germline genetic variation, on susceptibility to breast cancer overall and for disease subtypes characterized by clinical and molecular markers. Define the influence of risk factors and tumour subtypes on clinical prognosis. Develop, validate and implement breast cancer risk and prognostication models for breast cancer, overall and for different subtypes. Raise awareness; that is, promote the development and integration of personalized breast cancer prevention within national public health programmes	EU Horizon 2020	^29^
BCAC	Breast Cancer Association Consortium	International consortium of collaborative groups that share data from multiple studies in breast cancer. Identify genes that might be relevant to the risk of breast cancer. Provide a reliable assessment of the risks associated with these genes	Cancer Research UK	^81^
BRCA-ERC	Understanding cancer development in *BRCA1*/*2* pathogenic variant carriers for improved Early detection and Risk Control	Understand cell non-autonomous factors in carriers of *BRCA1* or *BRCA2* pathogenic variants that contribute to cancer development. Use cell-free DNA methylation-based markers for early detection of ovarian cancer. Develop new strategies and intermediate surrogate end points for non-surgical prevention of breast cancer	European Research Council	^31^
BRIDGES	Breast Cancer Risk After Diagnostic Gene Sequencing	Identify breast cancer susceptibility genes. Estimate risks associated with different genetic variants and incorporate into the BOADICEA risk-prediction model to provide individualized risk estimates. Implement individualized risk prediction in clinical settings	EU Horizon 2020	^30^
EU-TOPIA	Towards Improved Screening for Breast, Cervical and Colorectal Cancer in All of Europe	Develop and validate microsimulation models of breast, cervical and colorectal cancer screening in countries across Europe to assess current screening programmes. To assess inequalities in, and barriers to uptake of, screening. To develop road maps to improve existing screening programmes in Europe	EU Horizon 2020	^35^
FORECEE	Female Cancer Prediction Using Cervical Omics to Individualise Screening and Prevention	Utilize data on the cervical epigenome, genome and microbiome to develop personalized early detection and prevention strategies for breast, ovarian, endometrial and cervical cancer. Assess the ethical, health-economic, legal and societal aspects of using epigenetic markers for risk prediction. Develop strategies for communicating cancer risk	EU Horizon 2020	^32^
MyPeBS	My Personalized Breast Screening	Multicountry randomized trial of personalized breast cancer screening comparing risk-based screening to standard screening offered in each participating country among women aged 40–70 years^153^. Assess if individual risk-based screening is non-inferior or superior to the current standard of care in terms of reduction of the incidence of stage II or higher breast cancer	EU Horizon 2020	^33^
PERSPECTIVE I&I	Personalized Risk Assessment for Prevention and Early detection of Breast cancer: Integration and Implementation	Identification and validation of novel moderate to high risk breast cancer susceptibility genes. Improvement, validation and adaptation of a web-based tool for comprehensive breast cancer risk prediction that is suitable for the Canadian context. Development of a framework to support implementation of a personalized risk-based approach to breast cancer screening within existing mammography centres. Economic analyses for optimal personalized risk-based screening implementation	Canadian Institutes of Health Research, Genome Canada, Genome Quebec, Ontario Research Fund, Quebec Breast Cancer Foundation	^36^
PROCAS2	Predicting Risk of Cancer at Screening	Assess the feasibility of individualized risk assessment during screening appointments. Assess a range of effects of implementing personalized risk assessment on women, health-care staff and related organizations	National Institute for Health Research UK	^37^
WISDOM	Women Informed to Screen Depending on Measures of Risk	Multicentre, pragmatic, adaptive, preference-tolerant randomized controlled trial comparing risk-based screening to annual screening of women aged 40–74 years^152^. Determine if personalized breast cancer screening will lead to fewer harms, improve breast cancer prevention and be acceptable to women in comparison with standard annual screening	Patient-Centred Outcomes Research (PCORI)	^34^

To fulfil the promise of risk-stratified breast cancer prevention and screening, it is important not only to generate evidence on the individual component ‘jigsaw pieces’ of prevention and early detection programmes, but also to bring these pieces together in a complex adaptive system^39^. The European Collaborative on Personalized Early Detection and Prevention of Breast Cancer (ENVISION) comprises leading international research consortia working in this specific field (Table 1). In 2019, the ENVISION network organized a consensus conference to identify research priorities and recommend actions required to enable evidence-based risk-stratified prevention and early detection programmes for breast cancer (Box 1; Supplementary Table 1).

In this Consensus Statement, we review the current knowledge, explore the barriers and opportunities, and define key areas for the development and implementation of risk assessment, risk-stratified prevention and early detection programmes for breast cancer. As representatives of the ENVISION network, we also present herein the recommendations formulated at the 2019 consensus conference (Box 2) in the hope that they stimulate and guide such programmes.

Box 1 Process of developing the recommendations of the ENVISION network The European Collaborative on Personalized Early Detection and Prevention of Breast Cancer (ENVISION) network meeting was attended by 119 delegates from 19 countries: 14 countries in Europe (Austria, Belgium, Denmark, Estonia, Finland, France, Germany, Italy, the Netherlands, Slovenia, Spain, Switzerland, Sweden and the UK) as well as Israel, the USA, Canada, Malaysia and Australia. Together, the delegates brought diverse expertise in risk-based breast cancer research and health services (epidemiology, statistics, genetics, epigenetics, oncology, clinical genetics, pathology, gynaecology, radiology, surgery, primary care, public health, psychology, ethics, health economics, policy, screening services and health-care management), with representatives from academia, health-care organizations, industry (information technology support), politics (government representatives) and non-profit organizations (Europa Donna and the Association of European Cancer League).

The meeting was held over 3 days. During the first day, presentations covered the latest evidence (‘where we are now’) relating to breast cancer risk prediction, risk stratification for prevention, risk stratification for early detection at the population level and the implementation of such strategies. Each presentation was followed by a discussion session for the delegates to identify gaps in research (‘where do we want to be’). During the second day, through six workshops (focused on risk assessment, early detection, prevention, engaging stakeholders, health-care organization readiness, and ethical, legal and social implications (ELSI)), the delegates explored how to meet these gaps (‘how do we get where we want to be’). During the third day, named delegates, in coordination with the presenters, discussants and the facilitators of the workshops, presented recommendation for each of the 18 areas covered in the ENVISION meeting (genetic risk, epigenetic risk, classical risk factors, risk prediction, breast cancer subtypes, imaging, diagnostic tools for early detection, prevention, specific considerations in high-risk women, outcome, trial logistics, implementation, economic evaluation, communication and decision aids, policy landscape, ELSI, workforce training and health-care organization readiness). The presentation of each recommendation was followed by discussion and checking consensus.

Each delegate who contributed through presenting the evidence, the workshop discussions and the recommendations presented a written summary. After collating these summaries, all 119 delegates were asked for their feedback.

Box 2 Summary of the key recommendations of the ENVISION ***Assessment of breast cancer risk***

- Risk-assessment tools should be validated using prospective cohorts in the context in which they will be used and for each population ancestry.
- Risk-assessment tools that enable better predictions of breast cancer subtype-specific risk and risk in women of non-European descent need to be developed and validated.
- Discovery research to identify additional genetic variants and new markers is required to improve risk stratification.
- The trade-off between the accuracy of comprehensive models and their usability at the population level should be evaluated.
- Algorithm transparency should be ensured, with explicit reporting of the assumptions made.

***Breast cancer prevention***

- Ways to better select high-risk women predisposed to breast cancer of poor prognosis need to be developed.
- Clinically relevant surrogate markers (reflecting the field defect in breast tissues) that provide early indications of the effectiveness of the preventive measures in reducing incidence of breast cancer of poor prognosis need to be identified.
- Programmes should incorporate healthy lifestyle recommendations for women at all risk levels.
- Prevention-specific drug doses, schemes and schedules need to be defined, and rational drug repositioning strategies should be explored.
- Better and early assessment of the acceptability of new preventive interventions is required.

***Risk-stratified early detection***

- Discovery research is required to identify and validate early detection markers that can differentiate progressive from non-progressive breast cancers.
- Develop risk-stratified early detection strategies underpinned by understanding of how the natural course of breast cancer, sensitivity of the test (for example, mammography) and the probability of overdiagnosis vary according to risk levels.
- Optimize variables related to risk assessment (which risk factors to include, what age to start screening, how often to screen, and so on) and risk stratification (how many risk groups to specify and the risk threshold for each group), thus resulting in a cost-effective, feasible, acceptable and equitably accessible early detection programme.
- Modelling studies can be used to inform on long-term population outcomes and the optimal design of risk-stratified early detection programmes.
- Pragmatic randomized study designs, such as randomized health service studies, should be used to generate evidence on the effectiveness of risk-stratified early detection approaches in a given setting.

***Programme implementation***

- Adopt hybrid effectiveness–implementation research designs to reduce the time lag between generation of evidence on the effectiveness of a programme and its implementation.
- Shift away from small studies with hypothetical scenarios performed in silos to multidisciplinary research with engagement of all stakeholders to ensure a systems approach to implementation studies in real-world settings.
- A framework for a learning health-care system should be adopted.
- The implementation process in a given setting needs to be aligned with health-care organization readiness for change and the social values, preferences and norms.
- The best ways of communicating risk and supporting behavioural changes in response to risk information need to be identified.

## Risk assessment for breast cancer

### Established risk factors

Breast cancer risk can be predicted using a combination of common genetic variants, mostly single-nucleotide polymorphisms (SNPs); rare coding variants of susceptibility genes, including *BRCA1/2*, *PALB2*, *CHEK2* and *ATM*; mammographic breast density; benign abnormalities in breast biopsy specimens; hormonal, anthropometric and lifestyle factors; family history of the disease; and, potentially, epigenetic markers^11,13,40–43^. Genome-wide association studies (GWAS) have resulted in the identification of >180 independent common genetic variants that together account for ~20% of the familial relative risk of breast cancer and ~40% of the heritability attributed to all common variants on genome-wide SNP arrays^40,41^. Each variant confers a small risk, but their effects can be combined into polygenic risk scores (PRSs) that are predictive of the risk of developing breast cancer, thereby enabling breast cancer risk stratification in the general population^44–46^.

The performance of current PRSs has been thoroughly validated in European populations^44^. The relative risks associated with individual SNPs and PRSs vary between breast cancer subtypes, with oestrogen receptor-positive (ER^+^) disease being more strongly predicted than other forms of the disease^40,41,44^. The current best performing PRS is based on 313 SNPs (PRS_313_): women in the highest 1% of the risk distribution have an approximately fourfold and threefold greater risk of developing ER^+^ and ER^−^ breast cancers, respectively, compared with women in the middle quintile (40–60th percentile)^44^. The risk reflected in the PRSs seems to be independent of other established risk factors — that is, the effects are approximately multiplicative^43^. PRS_313_ provides the highest level of breast cancer risk stratification in the population, followed by mammographic breast density and the other risk factors^45,47^.

Protein-truncating variants (PTVs) in approximately 12 genes are associated with breast cancer risk^42,48^; for some, the strength of association has been demonstrated to differ between ER^+^ and ER^−^ disease^49,50^. The risk estimates for PTVs of some genes are, however, very imprecise (Table 2). Missense mutations in a subset of these genes have also been associated with an increased risk of breast cancer^42,51–53^. Evidence from in silico and functional studies can help to define this subset of cancers with non-truncating variants^54–56^. For rare individual variants associated with risk, however, the level of risk that they impart remains uncertain. Most genes tested using commercial multigene panels have not been systematically investigated as breast cancer susceptibility genes. The Clinical Genome Resource (ClinGen) framework has assessed the strength of evidence between selected putative susceptibility genes and breast cancer and established definitive clinical validity classifications for only 10 of 31 genes commonly tested when evaluating breast cancer risk^57^ (Table 2).

**Table 2:** Genes with rare variants associated with an increased breast cancer risk

Gene	PTV associated with breast cancer risk	Missense variants associated with breast cancer risk	Relative risk for PTV (90% CI)	Clinical Genome Resource (ClinGen) definition of clinical relevance
*ATM*	Yes	Yes	2.8 (2.2–3.7)	Definitive
*BARD1*	Likely	Unknown	2.1 (1.5–3.0)^48^	Definitive
*BRCA1*	Yes	Yes	11.4 (NA)	Definitive
*BRCA2*	Yes	Yes	11.7 (NA)	Definitive
*CDH1*	Yes	Unknown	6.6 (2.2–19.9)	Definitive
*CHEK2*	Yes	Yes	3.0 (2.6–3.5)	Definitive
*NBN*	Yes	Unknown	2.7 (1.9–3.7)	Limited
*NF1*	Yes	Unknown	2.6 (2.1–3.2)	Not evaluated
*PALB2*	Yes	Unknown	5.3 (3.0–9.4)	Definitive
*PTEN*	Yes	Yes	8.8 (2.7–34.4)^48^	Definitive
*RAD51D*	Likely	Unknown	2.1 (1.2–3.72)^48^	Limited
*STK11*	Yes	Unknown	No reliable estimate	Definitive
*TP53*	Yes	Yes	105 (62–165)	Definitive

Data were sourced from Easton et al.^42^ and Lee et al.^57^, with risk estimates derived from Easton et al.^42^, except where indicated otherwise. Note that risk estimates calculated by LaDuca et al.^48^ come with 95% confidence intervals (CIs) and are derived from a study of individuals referred for testing and, therefore, might not be unbiased estimates of the general population risk. NA, not available; PTV, protein-truncating variants.

### Emerging risk factors

The epigenome consists of various ‘layers’, including non-coding RNAs, histone modification and DNA methylation, and has an essential role in establishing the identity and function of any given cell by determining which genes remain silent and which are transcribed. A plethora of changes in DNA-methylation patterns have been described in breast cancers, and several of these changes are often also present in the non-malignant breast tissue adjacent to the cancer^58^, supporting the principle that an epigenetic field defect renders cells of these tissues susceptible to malignant transformation^59^. In addition to genetic background^60,61^, a large variety of non-genetic factors, including age^62^ and endocrine disruption^63,64^, that are known to modulate breast cancer risk also alter patterns of DNA methylation. On the basis of these insights, one might speculate that epigenetic profiles could predict breast cancer risk.

To date, several groups have attempted to develop epigenetic risk classifiers for breast cancer but with only modest success, which could be due to several reasons^65^. First, the vast majority of the studies to date used only blood samples for DNA-methylation analyses. Blood is readily available from participants of several large cohort studies^61,66^, but breast cancer is, by definition, an epithelial disease, and hence immune cells in the blood might not be an appropriate surrogate tissue for those of the breast. Second, unlike in germline genetic analyses, the timing of the sample collection for epigenetic analyses is crucial. For example, epigenetic analyses using samples obtained from women during cancer treatment are likely to produce results that reflect treatment effects and not cancer predisposition. Third, unlike PRSs, which are established by combining individual SNPs with risk associations that remain statistically significant after multiple test adjustment, epigenetic risk signatures are reflective of cell programmes; therefore, approaches that a priori select a large number of CpGs for inclusion in the epigenetic signatures are more likely to be appropriate. Fourth, the presence of a cancer can modify the epigenome of a particular surrogate tissue. For example, a higher granulocyte to lymphocyte ratio is detected in the blood of patients with ovarian cancer, which subsequently alters the DNA-methylation signature observed when assessing peripheral blood mononuclear cells^67^. Thus, validation of risk-predictive signatures in population-based cohorts is important; however, the majority of the existing cohorts do not have appropriate samples available (owing to non-standardized collection, storage conditions and times, and so on), which makes this validation process prone to producing false-negative results.

Nevertheless, DNA-methylation signatures in easy-to-collect surrogate tissues hold promise, not only in advancing risk-prediction strategies, but also, of equal importance, in providing novel opportunities to monitor the effects of cancer-preventive measures. In addition to epigenetic markers, serum levels of steroid hormones^68–70^ and a double-strand DNA break-repair phenotype of peripheral blood cells^71,72^ have substantial potential to identify women with a high risk of developing breast cancer.

### Risk-prediction models

Several breast cancer risk-prediction models are available. Empirical models such as the Gail model^73^, the Breast Cancer Surveillance Consortium (BCSC) risk calculator^74^ and the Individualized Coherent Absolute Risk Estimator (iCARE)^75–77^ do not consider explicit genetic models of inheritance and are primarily intended for use in women in the general population. By contrast, genetic models such as Tyrer–Cuzick^78^ and BOADICEA^45,79^ can, in principle, accommodate detailed family history information (including the exact pedigree structure and information on distant relatives) and can, therefore, be applied both at the general population level and in women with a strong family history of breast cancer. These models all vary in terms of the risk factors considered, the study designs and types of data used in their development, and their analytical methods. The validity and clinical utility of these risk-assessment tools must be demonstrated before they are implemented routinely in the clinical setting^80^.

#### Validity

Analytical validity refers to the accuracy of the test in measuring the underlying genotypes (for example, through gene-panel testing or sequencing assay for rare mutations), PRSs (for example, using SNP-genotyping technologies) and other lifestyle and hormonal risk factors (which can be self-reported or available through electronic health records). Importantly, the analytical validity of comprehensive breast cancer risk-prediction models also depends on having reliable relative risk estimates for the effects of the various risk factors; having precise risk estimates of the associations with individual rare and common genetic variants; as well as estimates of the joint effects of common genetic variants, the joint effects of common and rare genetic variants, and the combined effects of genetic and other risk factors, including a family history of cancer. Clinical validity refers to the accuracy of the tool in predicting the occurrence of breast cancer.

Ideally, the individual and combined associations of the various risk factors should be derived from large, well-designed cohort studies that are representative of the population in which the models are intended to be applied. However, cohorts with data that include information on all known risk factors are not widely available; therefore, synthetic mathematical approaches have been developed that combine the risk-factor distributions from separate cohorts^45,75,76^. Data generated by the B-CAST^29^, BRIDGES^30^, BCAC^81^ and CIMBA^82^ consortia (Table 1) provide a platform for estimating the individual and combined risk-factor distributions and breast cancer risk and have been used in the development of the iCARE^77^ and BOADICEA^45^ breast cancer risk-predication models. Some empirical models, which are commercially available, have been modified to incorporate breast cancer PRSs, but without accounting for the fact that PRSs explain a large fraction of the familial relative risk of breast cancer. The failure to adjust these models to account for family history of breast cancer results in substantial levels of miscalibration in different risk categories and subsequently compromises the clinical validity of the model^46^.

With regard to clinical validity, several validation studies assessing model calibration (that is, the agreement between the predicted and the observed risk) or discrimination (the ability of a risk score to discriminate between those who will and those who will not develop the disease) in large independent cohorts have been published^83,84^. The interpretation of the literature is challenging, however, because these studies have not necessarily assessed both model calibration and discrimination in the same sample. Moreover, head-to-head comparisons of risk models using the same datasets are lacking. Often the published validation studies have used older versions of the risk models without data on all model components (in particular, mammographic breast density), have limited sample sizes and have varying timescales over which predictions are made, which depend on the follow-up duration of the study.

Ongoing studies by B-CAST^29^ and BRIDGES^30^ aim to address these issues by evaluating risk-assessment models in multiple prospective cohorts of women initially without breast cancer in diverse settings. Preliminary results indicate that the iCARE^77,85^ and BOADICEA^45^ models have well calibrated categories of predicted risk and discriminate well between women who develop breast cancer over 5–10 years of follow-up study from those who do not^84^. As such, these models provide valid risk-prediction tools that can be used in clinical practice.

#### Clinical utility

**Fig. 2:**
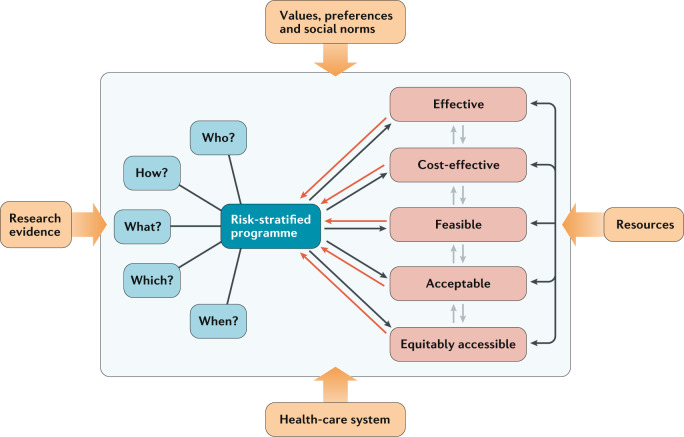
Risk-stratified early detection and prevention programmes as complex adaptive systems. Various questions will define the risk-stratified programme, including which risk factors to include in risk assessments, what risk threshold to use for risk stratification, how many risk groups to have, when to do risk assessments, how often to screen and to whom screening should be offered, as well as which interventions should be used in individuals deemed to be at high risk. Decision-making regarding these questions will be influenced by the research evidence, the available resources, the health-care setting and societal values, preferences and social norms. The choices made in addressing each of these questions will determine whether the programme will be effective in reducing cancer-specific death and improving the benefit–harm balance of screening and be cost-effective, acceptable, accessible and feasible to implement. Dynamic interactions exist between each of these factors, and thus a change in one factor affects all others. Hence, the importance of a holistic, ‘systems thinking’ approach.

Conceptually, clinical utility refers to the usefulness, benefits and harms of an intervention^86,87^. Clinical utility is a multidimensional construct covering effectiveness and cost-effectiveness, as well as the psychosocial, ethical and legal implications of an intervention^86^. Risk assessment per se does not have inherent clinical utility; the subsequent adoption of a risk-based intervention based on the results of the assessment is what influences the health outcomes^88^. The use of such a strategy depends on whether the risk-based intervention is appropriate, accessible, practicable and acceptable^86^. The interactions of these factors and challenges in assessing them are discussed in more detail in later sections of this article (Fig. 2).

### Future directions in risk prediction

We have identified several key areas for development in breast cancer risk modelling (Box 2). These research priorities include models that better predict the risk of specific subtypes of breast cancer and with improved risk stratification of women of all ancestries, particularly non-European ancestries, who have been understudied to date.

Subtyping of breast cancer is currently used routinely in prognostication and treatment, although its use in the context of prevention and early detection of the disease is limited. The ability to predict susceptibility to the typically more aggressive, ER^−^ forms of breast cancer would enable selection of women for enhanced surveillance. Better datasets containing both clinical and genetic data are essential to develop and validate models that can more accurately predict subtype-specific risk, pathobiological behaviour and clinical outcomes. For example, B-CAST^29^ and BRIDGES^30^ are developing such data sources that integrate genetic, epidemiological, pathological and clinical data.

Multiancestry GWAS and targeted DNA-sequencing data from individuals of various ethnicities will enable translation of PRS-based and gene-based risks to populations of non-European ancestry. Heritability analyses indicate that breast cancer is a highly polygenic disease, with thousands of variants conferring a small effect on risk, and that larger studies would result in new discoveries^89^. The Confluence project^89^ aims to build a dataset comprising >300,000 patients with breast cancer and 300,000 individuals without the disease in order to conduct a multiancestry GWAS. This study will enable better understanding of the aetiology of distinct breast cancer subtypes, more powerful modelling of the underlying polygenic risk and improve risk stratification across groups of women with different ancestries.

A large fraction of the unexplained heritability of breast cancer might be attributable to rare variants (allele frequency <0.1%) not captured on SNP arrays^90^. Exome sequencing and replication studies with large cohorts, such as those being conducted by BRIDGES^30^ and PERSPECTIVE I&I^36^, should be informative in determining whether additional susceptibility genes, with risk-defining coding variation, exist. For non-protein-coding variants, however, much larger whole-genome sequencing datasets, coupled with genomic risk prediction (genotype imputation), will be required.

Other promising approaches to improve breast cancer risk prediction include imaging and blood-based biomarkers. Improved use of mammography or MRI to predict risk is a particularly attractive area of research^91–93^; parenchymal textual features beyond simple mammographic breast density, such as the co-occurrence matrix and multiresolution spectral features, have been shown to be important^94^ and might be independently predictive of the development of breast cancer^92,95,96^. Screening programmes provide longitudinal data that can facilitate studies to identify such imaging biomarkers. Potential blood-based biomarkers include microRNAs, tumour-educated platelets and circulating tumour DNA^97–99^. However, these markers might be more suitable for short-term early detection than long-term risk prediction (since they are perhaps more likely to reflect the presence of cancer rather than cancer susceptibility), and large longitudinal collections of samples will be required to study them.

Comprehensive models incorporating genetic and epidemiological risk factors and mammographic breast density enable more accurate risk stratification in the general population, as well as in carriers of germline pathogenic variants, than is possible with models that consider only PRS^45,47^. Repeat collection of information on the non-genetic risk factors at a population level raises further complexities in the logistics of risk assessment. The feasibility, clinical utility, costs and cost-effectiveness of risk-based programmes using a comprehensive model versus a model with only PRS need to be evaluated.

To enhance the credibility of a given model, and thus confidence in the results, transparency (that is, a clear description of the model structure, equations, parameter values and assumptions) and validation in relevant settings are essential. The challenge lies in having a consensus on the criteria for sufficient evidence to declare a model as ‘valid’ for a particular application^100^.

## Risk-stratified prevention

In high-income countries that have implemented strategies to prevent or mitigate cardiovascular disease (CVD), cancer has superseded CVD to become the most common cause of death^101^. In the context of CVD, clinical parameters indicative of risk (for example, blood pressure and serum lipid levels) can be successfully targeted and subsequently used to monitor preventive actions^102^. However, mirroring these concepts in the context of cancer has not been possible to date. Cancer development is a multifactorial process that occurs at various stages of life and sometimes decades in advance of diagnosis. Avoiding certain risk factors for breast cancer (for example, hormone replacement therapy, particularly those containing progesterone^103^), as well as adopting healthier lifestyle patterns (such as limiting alcohol consumption^104,105^ and maintaining a healthy weight^106^), can have long-term cancer-preventive effects. Nevertheless, many of the risk factors for breast cancer (including a family history of the disease and genetic predisposition, birthweight, age at menarche, age at first live birth and age at menopause) are not modifiable, and in many cases the biological mechanism underlying the associated increase in breast cancer risk remains unknown. Notwithstanding, several active strategies have been shown to modify breast cancer risk.

### Chemoprevention with anti-oestrogens

The results of prospective randomized controlled trials (RCTs) evaluating primary prevention of breast cancer using selective oestrogen receptor modulators or aromatase inhibitors have consistently shown a reduced incidence in hormone receptor-positive subtypes of the disease^107–119^. However, in order to prevent one breast cancer in the next 20 years, 22 women needed to take tamoxifen daily for 5 years^117^. The considerable adverse effects of anti-oestrogens and the fact that none of these trials has shown any overall or breast cancer-specific survival benefits or a reduction in the incidence of aggressive, hormone receptor-negative forms of breast cancer make it difficult to judge whether treating healthy women with these drugs is a more effective strategy than reserving them for the adjuvant treatment of only those who actually develop breast cancer. Nevertheless, the US Preventive Services Task Force have judged that serious adverse effects, such as thrombosis and endometrial cancer, are uncommon and the more common toxicities, such as vasomotor symptoms, are reversible and were only marginally more frequent in women on active treatment than in those receiving placebo in the aforementioned RCTs^120^. Accordingly, several international guidelines recommend the use of anti-oestrogens as chemopreventives for women at increased risk of breast cancer^16,121^. Whether improved risk stratification would reduce the number of healthy women who need to take anti-oestrogens in order to achieve the same preventive effect will need to be established in future RCTs.

### Surgical prevention

Prophylactic bilateral mastectomy is certainly the most effective way of preventing breast cancer and reducing breast cancer-specific deaths in the small minority of women (perhaps 3%)^122^ with a germline pathogenic *BRCA1/2* variant^123^. Nipple-sparing mastectomies are a safe option for these women, with no known detriment to the risk reduction^124^. General complications include wound dehiscence, infection, implant loss or flap necrosis, asymmetry and capsular contracture^125^. For nipple-sparing mastectomies, the overall complication rate has been reported to be 22.3%, and the rate of nipple necrosis was 5.9%^126^. However, surgery can be associated with other complications and adverse effects, including psychological distress with body image change, and has implications relating to resources. Thus, clinical utility, feasibility and acceptability need to be evaluated in order to set the risk threshold for surgical intervention.

### Other preventive strategies

In past few years, several new targets of potential preventive interventions for breast cancer have been discovered. In particular, progesterone has an essential role in the development of aggressive breast cancers. A meta-analysis of 58 studies revealed that women receiving a progesterone-containing menopausal hormone therapy not only have a higher incidence of breast cancer than women not receiving such therapy or those receiving oestrogen-only treatments, but also more cancers that had spread beyond the breast^103^. Furthermore, the data indicated that women receiving progesterone-containing therapy are more likely to die from breast cancer than women treated only with oestrogens^127^. Additional evidence for the role of progesterone in breast carcinogenesis comes from the observation that women with germline pathogenic *BRCA1/2* variants have elevated levels of luteal phase progesterone compared with those observed in carriers of non-pathogenic *BRCA1/2* variants^128^. This increase in progesterone levels leads to an increase in receptor activator of nuclear factor-κB ligand (RANKL) levels in the breast^129–133^, as well as reduced levels of the physiological RANKL antagonist osteoprotegerin^129^. These effects in turn lead to an expansion of ER^−^ and progesterone receptor-negative mammary stem cells and eventual breast cancer formation^134^. In mouse models, *Brca1/2*-mediated breast cancer formation can be prevented by disrupting the progesterone signalling pathway using the competitive progesterone receptor antagonist mifepristone^135^. In addition, the findings of a case–control study involving women with germline *BRCA1/2* mutations indicate that moderate use of dietary supplements containing folic acid and vitamin B12 can be protective against *BRCA1/2*-associated breast cancer^136^. Other potential risk-reducing chemotherapeutics include aspirin, metformin, statins or other agents^137^.

To date, clinical trial evidence supporting these chemoprevention strategies is lacking. Denosumab, a fully humanized antagonistic monoclonal antibody targeting RANKL, has been shown to reduce breast epithelial cell proliferation in three premenopausal volunteers^134^. In postmenopausal women with breast cancer, however, denosumab does not seem to alter the incidence of contralateral breast cancer^138^. A prevention study of this agent in carriers of pathogenic *BRCA1* variants is underway^139^.

### Future directions in prevention

Several challenges need to be addressed to advance the field of breast cancer prevention. First, drugs that can reduce the incidence of aggressive breast cancers, for example, of the triple-negative, HER2^+^ or luminal B subtypes, need to be identified.

Second, the required doses and frequency of administration of these potential preventive drugs need to be established. Unlike tamoxifen and aromatase inhibitors, the efficacy and safety of which have been tested in many thousands of women in the adjuvant treatment setting, no such data exist for the most promising novel preventive drugs (that is, progesterone antagonists and denosumab).

Third, efforts are needed to develop an effective approach to selecting women for whom breast cancer primary or secondary prevention measures will provide survival benefits. None of the current risk-prediction models intended to identify women at an increased risk of developing breast cancer in the absence of a familial predisposition (that is, mainly carriers of pathogenic *BRCA1/2* variants) selectively identifies those women at risk of developing an aggressive cancer that, if not prevented, would likely lead to death.

**Fig. 3:**
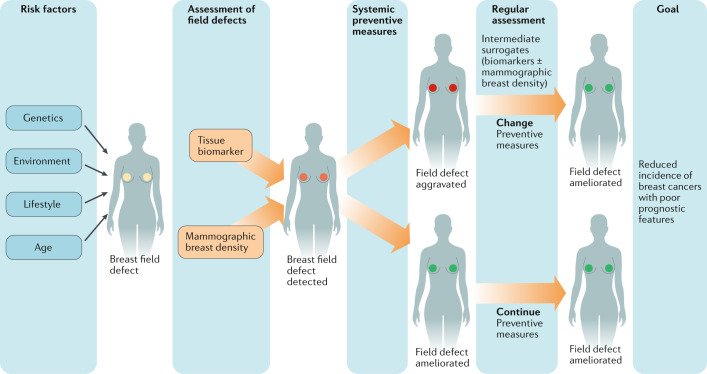
Overview of personalized risk reduction and breast cancer prevention paradigms. Various risk factors contribute to field defects in breast tissues that favour the development of breast cancer. The presence of such field defects can be assessed using biomarkers and/or imaging to guide personalized prevention strategies, the success of which can be monitored on an ongoing basis through intermediate surrogates (for example, reduction or resolution of the field defect) that reflect the ultimate goal of a decreased incidence of breast cancers with features indicative of a poor prognosis.

Fourth, surrogate end points are required (Box 2). Demonstration of a reduction in breast cancer-related mortality is recommended before implementation of any early detection strategy^140^ whereas, for prevention strategies, robust evidence of a reduced cancer incidence seems to be sufficient to recommend clinical implementation^141^. The focus should not, however, be a reduction in the incidence of any breast cancer, but rather of breast cancers that hold a poor prognosis. Intermediate surrogate markers are urgently required to enable timely assessment of the efficacy of potential new breast cancer-preventive drugs, particularly in women at high risk of the disease so as not to substantially delay or preclude bilateral mastectomy that is a safe risk-reducing option. A reduction in mammographic breast density has proved to be an excellent predictor of response to tamoxifen in the preventive setting^142^. In addition, molecular biomarkers, assessed directly in breast tissue and reflective of a field defect^58^ or indirectly in a surrogate tissue or blood^32^, could potentially provide three essential advantages in prevention strategies for premenopausal women at high risk of breast cancer: 1) they can be measured frequently; 2) the dynamics of the molecular biomarkers in individual volunteers might reflect the cancer risk in real time, and thus individual adjustments to preventive measures could be made ad hoc; and 3) unlike many imaging-based markers, they do not require repeated exposure to x-rays (Fig. 3).

Finally, strategies should be developed to increase the acceptability and accessibility of interventions used for breast cancer prevention. Notably, the efficacy of weight loss programmes has been shown to be greater among individuals who are aware of being at high risk of developing breast cancer^143^. Importantly, weight loss^144^ and regular exercise^145,146^ not only decrease breast cancer risk but also the risks of other cancers and CVDs. Considering the general health benefits, lifestyle interventions could be recommended to women at all levels of breast cancer risk^147^. Thus, developing effective ways to make both lifestyle and chemoprevention options widely available (including within screening programmes), acceptable and better understood by health-care professionals and the public is essential^148^ (Box 2).

## Risk-stratified early detection

The Cancer Control Joint Action European Guide on Quality Improvement in Comprehensive Cancer Control^149^ recommends that the benefits (cancer-specific deaths averted and quality-adjusted life years gained), harms (related to false screen findings and subsequent investigations, and overdiagnoses and the associated treatments) and cost-effectiveness of a screening programme should be estimated to guide decisions on implementation. RCTs should be used to generate the primary evidence on the effectiveness of a new screening programme in reducing cancer-specific mortality^149^. When modifying currently running programmes, however, questions remain regarding what constitutes supportive evidence (that is, the required level of evidence and study design)^150^ and how complete the evidence needs to be before recommendations for implementation can be made^151^.

### Effectiveness

Two short-term RCTs evaluating the effectiveness of risk-stratified screening for breast cancer are currently ongoing: WISDOM in the USA^152^ and MyPeBS in Europe^153^. While the two trials share a similar design, with intermediate outcome measures (such as stage distribution) as end points, their protocols are adapted to the local health-care settings.

WISDOM^152^ is a multicentre, pragmatic, adaptive, preference-tolerant RCT comparing risk-based screening to annual screening in women aged 40–74 years. WISDOM is designed to determine whether risk-based screening is as safe as annual mammographic screening (number of stage ≥IIB cancers is no higher than that observed with annual screening), but with less morbidity (measured according to the number of breast biopsies performed) as well as greater acceptability, conductivity to preventive interventions and health-care value^152^. Women in the risk-based screening arm are receiving a personal risk assessment based on the BCSC risk calculator integrated with a PRS (which has been adapted during the course of the trial) and testing of a panel of nine susceptibility genes^154^. Those women are being stratified into four risk groups: highest risk, elevated risk, average risk and lowest risk. Each group is recommended a screening strategy that varies in starting age and the frequency and modality of screening — annual mammography with adjunctive MRI, annual mammography, biennial mammography and deferred screening until the age of 50 years (in the lowest risk group comprising women aged 40–49 years with 5-year absolute risk <1.3%), respectively^155^.

MyPeBS^153^ is a pragmatic, multicentre RCT that is being performed in five countries (Belgium, France, Israel, Italy and the UK) to determine if risk-based screening of women aged 40–70 years is non-inferior, in terms of the 4-year incidence of stage ≥II breast cancer, to the standard screening programme currently offered in each participating country (screening every 2–3 years beginning at 40–50 years of age and ending at 69–74 years of age). In MyPeBS, the frequency and modality of screening vary according to the level of risk predicted using PRS_313_ combined with the BCSC^74^ or the Tyrer–Cuzick^78^ risk calculator. The latter calculator is used only in women with more than one first-degree relative with a history of breast or ovarian cancer. In MyPeBS, women are also being classified into four risk groups^153^, although the risk thresholds differ from those used in WISDOM. However, the lead investigators of both trials are ensuring that data are collected in a similar way and have committed to pooling the data to improve the ability to learn from each study.

RCTs of screening interventions provide the strongest evidence of efficacy, although they have certain limitations. In particular, lifetime health effects cannot be observed in RCTs with limited follow-up durations. Thus, the observed benefit–harm trade-offs might not accurately reflect those expected with long-term population screening^156^. Moreover, the outcomes of screening depend on the screening strategy (including the choice of risk-assessment tool, risk thresholds, screening modalities, screen intervals and starting and stopping ages) and variables relating to the setting (such as the available infrastructure, levels of adherence and population preferences)^149^. Variations in any of these elements can alter the benefit–harm trade-offs. Finding the optimum strategy for a given population requires comparisons of several alternative screening strategies; however, RCTs — particularly of screening strategies that require very large cohorts and long follow-up durations — are inherently limited in their ability to compare more than a few approaches (typically two or three).

Simulations using natural history models and decision analysis models constitute useful tools to study the long-term benefits and harms as well as the cost-effectiveness of various screening strategies^157–159^. Such modelling studies can precede or follow RCTs of screening interventions. Lifetime health effects can be modelled using empirical data — for example, from RCTs of different approaches to screening, long-term observational studies and clinical registries^160^. Modelling studies that incorporate data on the population structure and preferences, the natural history and prevalence of disease, life expectancy, the available resources and costs can provide an indication of which screening strategies are likely to be optimal in a given setting^160^. Thereafter, the most promising strategies could be tested in RCTs. Thus, modelling studies can inform population-screening policies by extrapolating evidence beyond the time horizon of prospective trials and enabling the translation of evidence from one study population to another.

To date, evidence on the effectiveness of risk-stratified screening has come from model-based studies^26,27,161^. Modelling approaches have limitations, however. Models present a simplified representation of disease progression and intervention outcomes. Moreover, the accuracy of the results of modelling is dependent on the underlying assumptions and the degree of uncertainty in the input parameters^162^. Estimating overdiagnosis through simulations is particularly challenging^163^ and more so in the absence of data on the rates of disease progression for different risk groups.

### Cost-effectiveness

Thus far, few studies have evaluated the cost-effectiveness and benefit–harm trade-offs of risk-stratified screening for breast cancer. Vilaprinyo et al.^161^ risk stratified women using several combinations of risk factors and showed that quinquennial or triennial screening for the low-risk or moderate-risk groups and annual screening for the high-risk group, from 50–74 years of age, would reduce costs, the number of false-positive findings and overdiagnosis, while averting the same number of deaths as biennial screening between the ages of 50 and 69 years. Trentham-Dietz et al.^27^ used a combination of mammographic breast density and four exemplar relative risk levels for risk stratification and showed that triennial screening of average-risk women with low breast density, starting at 50 years of age, and annual screening of higher-risk women of the same age with high breast density would be cost-effective at a threshold of $100,000 per quality-adjusted-life years gained and would maintain a similar or better balance of benefits and harms than biennial screening of average-risk women. Pashayan et al.^26^ used the distribution of polygenic risk in the population combined with other risk factors for stratification and showed that, compared with screening women from age 50–69 years triennially, not screening women at lower risk of developing breast cancer would improve the cost-effectiveness and benefit to harm ratio of the breast-screening programme.

### Policy implications

When modifying an existing breast cancer screening programme, several considerations need to be taken into account. In particular, agreement should be reached on the framework of expected changes and acceptable trade-offs, whether in benefits, harms, net benefit, equity, cost or opportunity cost, in order to facilitate decisions on whether the evidence is supportive of the adapted programme. The ultimate aim is to implement risk-stratified screening that is justifiable from ethical, legal and societal viewpoints.

The policy priorities should be explicit: is the priority to maximize the return on investment or maximize the benefits of screening? That is, will the total number of screens and/or the budget allocated to the screening programme stay the same, but be utilized in a different way to maximize the benefits by focusing on higher-risk groups; or will the screening efforts and resources be increased to enable tailoring of screening to the risk level of each individual?

### Future directions in early detection

We have identified several key areas for future research to improve early breast cancer detection (Box 2). The evidence from modelling studies indicates that risk-stratified screening approaches could potentially improve the efficiency and the benefit–harm balance of breast cancer screening programmes. Further data are required, however, on how the natural course of breast cancer, the sensitivity and specificity of mammography, as well as the probability of overdiagnosis vary according to the underlying risk of the disease. This information is needed to minimize the assumptions and uncertainties in the estimates used in models of risk-tailored screening strategies.

To have confidence in the validity of the outputs of modelling studies, the models have to be well calibrated, the structural assumptions and parameter estimates should be reported clearly and explicitly and the effects of alternative assumptions should be assessed in sensitivity analyses^100,164–166^. Having the code made open-source and easily accessible will enhance the transparency of the model^158^.

In countries with existing breast cancer screening programmes, randomized health service trial designs could be used to evaluate risk-based screening in routine health-care settings. Such trials enable the comparison of a new policy or intervention to the current standard approach within the context of an existing health service^167^. Indeed, although modelling, routine monitoring and observational studies can provide helpful evidence, they are not a replacement for randomized health service studies^167^.

Trading off benefits and harms of different screening strategies is a fundamentally value-laden activity. Discrete choice experiments (DCEs) provide a quantitative approach to eliciting women’s preferences^168^. In a DCE, participants are asked to choose between a series of alternative hypothetical scenarios described in terms of characteristics (or attributes) of the approach and associated levels of, for example, benefit and/or harm. In making these choices, participants are trading off between preferred and less preferred attributes presented in each alternative scenario. Incorporating the choice probability derived from DCEs for each screening approach into decision analytical modelling might facilitate the identification of optimal screening strategies.

In addition to cost-effectiveness analyses, budget-impact analyses will be needed to assess the affordability of a risk-stratified screening programme in a given setting^169^. Finally, although risk-stratified screening could potentially reduce overdiagnosis, a major need remains for tests that can differentiate, at diagnosis, tumours with progressive potential in order to reduce overtreatment. At present, no test is available for such differentiation at diagnosis. Biomarker-driven decisions regarding adjuvant therapy have, however, been incorporated into guidelines for the management of women with certain types of breast cancer^170^, which suggests that such an approach might be viable at diagnosis.

## Implementation

**Fig. 4:**
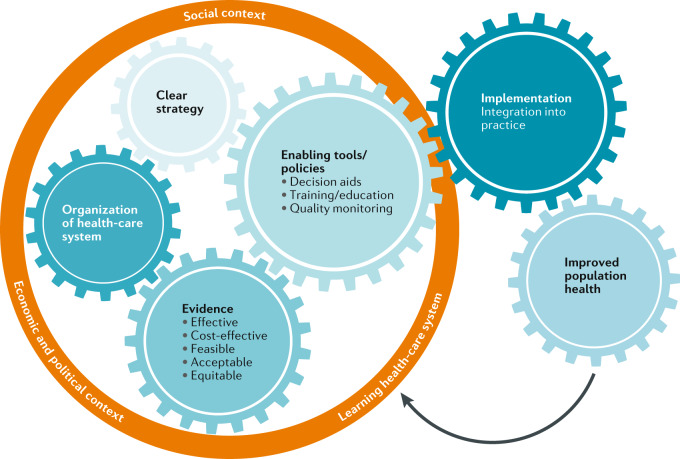
Implementation of risk-stratified early detection and prevention programmes in a learning health-care system. The schematic illustrates the various multilevel interactions between the different components needed for the implementation of risk-stratified programmes for the early detection and prevention of cancer. The ultimate goal is an improvement in population health outcomes. To achieve this goal, the process has to be iterative within a learning health-care system.

Before risk-stratified prevention and early detection programmes for breast cancer can be implemented, health-care providers and policymakers would need to plan the resources, build the infrastructure for population-wide risk assessment, develop policies and regulations to protect the public from stigmatization and discrimination, and provide support for informed decision-making of individual women regarding whether or not to participate in the screening programme. Ultimately, these actions are needed to ensure the feasibility and affordability of providing a high-quality risk-stratified screening programme that is accessible to all and is aligned with public values and preferences. There will not be a single predefined way of organizing and delivering such programmes. The optimal approach will be context-specific to account for the idiosyncrasies of the health-care system, as well as the social, economic, cultural and political context (Fig. 4). Here, we are not dealing with a mathematical or technical problem; the implementation of risk-adapted breast cancer prevention and screening strategies does not constitute a simple change that has a simple solution, but rather necessitates complex adaptive changes that require all stakeholders, scientists, health-care professionals, the lay public and policymakers to work together.

### Health-care organization readiness

Organizational readiness for systems change is widely recognized as being necessary for the successful implementation of complex changes in health-care settings^171^. This state reflects the extent to which those involved in implementing the new approach are primed, motivated and capable of achieving the required changes^172^. Organizational readiness is a dynamic process with pull and push factors between what is possible owing to constant emergence of new technological opportunities and what resources are available^173^.

To address the challenge of a constantly changing environment, health-care organizations should embrace an evolutionary approach, rather than espouse a sudden dramatic shift, by adopting a learning organizational culture and building on existing infrastructure^65^. In keeping with this concept, the adaptive design of WISDOM enables learning and adaptation of the risk-assessment model and the screening recommendations accordingly over the course of the trial, instead of waiting for certain new discoveries to emerge before starting the trial, or excluding participants of non-European ancestry (for whom limited relevant data are currently available)^152^. The coverage with evidence development (CED) model^174^ is a way of developing a ‘learning-based health-care system’. CED provides a mechanism for promising but unproven health technologies to enter practice sooner, through time-limited reimbursement that is conditional on a specific requirement for generation of further evidence on the performance of the new technology.

Readiness for change requires the commitment and engagement of all stakeholders, resources (including knowledge, skills, time, money and infrastructure) and governance^171^. To ensure the commitment of health-care organizations, the need for a change should be recognized and embedded in a shared vision, with leadership and coalition of all stakeholders^171,175^. To achieve a shared vision, the stakeholders have to agree on a framework of values that are aligned with those of the health-care organization. For example, health-care organizations value time-efficiency; therefore, successful implementation would require time-respecting strategies and tools, such as having one test to predict multiple cancers (which is a goal that FORECEE^32^ aims to achieve). Overall, vision, skills, incentives, resources and action plans are needed to achieve the systems change that will be required for implementation of risk-stratified prevention and early detection programmes for breast cancer^176^.

### Stakeholder engagement

Given the diverse opinions on breast cancer screening among key stakeholders at present and the specific challenges of risk-stratified screening, engagement of all stakeholders is crucial to implementation of new programmes. A stakeholder is a person, group or organization involved in or affected by a decision^177^. Key stakeholders in breast cancer prevention and screening include the users and the providers of the service, health-care professionals, policymakers, payers, advocacy groups, researchers and others. Stakeholder engagement would enable the identification of potential misunderstandings among the various stakeholders regarding opposition to, and perspectives on, the implementation of a risk-stratified programme^178^. Using a multistakeholder approach to reach agreement on what would constitute sufficient evidence to change practice and on guidelines would increase the chances of implementing the research findings within the health-care system^152^. Such an approach would also help to articulate the values and preferences of the wider community and to build mutual trust, thereby facilitating the implementation of a programme that is accessible and acceptable. Stakeholder analysis^179^ would be useful to not only identify the key stakeholders, but also their interests and influences, and the level of involvement of each (whether it be provision of information, consultation, deliberation, participatory decision-making or delegated decisions)^177^.

### Risk communication and its impact

Many women overestimate their risk of developing breast cancer^180^ and thus perceive screening as ‘almost always a good idea’^181^. This attitude is attributable to suboptimal levels of risk literacy among both patients and doctors as well as the limited transparency in the reporting of risks in the media and patient brochures^182^. Importantly, therefore, women should be transparently informed — for example, using fact boxes^183,184^ — about their baseline risks and the benefit to harm ratio of risk-based screening as compared to the existing options of a universal screening approach or no screening^185^. The development of risk-stratified programmes will need to include consideration of how to update risk assessments as risk-prediction models improve and how to communicate these changes to individuals.

Communicating information on breast cancer risk alone is unlikely to result in changes in health-related behaviours, such as smoking or low levels of physical activity^148,186,187^. Indeed, a methodical review of nine systematic reviews, encompassing at total of 36 unique studies, revealed no evidence that providing risk information would have strong, consistent or sustained effects on behaviour^186^. Changes in health-related behaviour can, however, be facilitated by including elements of interventions to alter the behaviour in question^143^.

Importantly, the available evidence suggests that providing women with their breast cancer risk estimates is unlikely to produce elevated distress^188^. Nevertheless, knowledge of whether providing risk estimates will promote informed choices regarding screening attendance is lacking, although the evidence base is starting to increase^148^. More definitive conclusions regarding the behavioural and emotional effects of receiving risk estimates require studies specifically designed to assess these questions (for example, PROCAS2 (ref.^37^), MyPeBS^153^ and PERSPECTIVE I&I^36^).

### Acceptability

Acceptability is a complex and poorly defined concept^189^. The level of uptake is one index of the acceptability of a risk assessment. Many studies have addressed the issue of acceptability of risk-stratified screening for breast cancer from the perspective of women^38,190^ and of health-care professionals and policymakers^191^. The available evidence suggests that risk-stratified screening is broadly acceptable to women if it involves the potential for more frequent screening for those deemed to be at high risk^192,193^.

By contrast, a number of concerns exist among professionals working in this area, not least regarding costs and the available evidence base^38^. Similarly, major reservations surrounding the appropriateness of reducing the frequency of screening for women deemed to be at low risk have been expressed by health-care professionals, policymakers and women themselves^194^. A few high-quality ongoing studies^36,37^ are examining these issues empirically, rather than discussing the issues as hypothetical possibilities^195^. Further research is needed to determine the feasibility of risk-stratified screening, particularly studies on implementation of screening in a research context, such as PERSPECTIVE I&I^36^ and PROCAS2 (ref.^37^).

### Workforce training

Effective delivery of risk-stratified prevention and screening services requires health-care professionals to be competent in the use of a risk tool, in interpreting and applying the risk scores and in communicating risk scores effectively to each individual, including discussion of the accuracy of the risk prediction and its future implications^196^. Risk-stratified approaches entail (epi)genetic testing for risk assessment. Health-care professionals need not become geneticists to effectively use the (epi)genomic information obtained^197^; however, they need to be sufficiently versed in (epi)genomics — for example, in understanding the contribution of common and rare coding variants to risk prediction, gene-panel testing and DNA-sequencing modalities and the implications of identifying pathogenic variants with poorly defined cancer risks or genetic variants of uncertain significance (VUS)^198^. Health-care systems should develop clear guidance related to the reporting of VUS in order to aid health-care professionals in the management of these variants, including descriptions of how patients with VUS should be informed if and when variants are found to confer an additional risk.

To engage with a new prevention and/or early detection scheme, the health-care professionals involved need to have a clear understanding of the rationale for risk stratification and risk-tailored interventions^196^, and have adequate knowledge of screening risk literacy^199^ and risk-communication skills; they should also have access to structured referral pathways for those women who need more detailed counselling. Accordingly, aspects of genomics and risk-stratified interventions should be integrated across the continuum of training for health-care professionals, from undergraduate education to broad specialty training to continuous professional development programmes. Educational-needs assessments should inform the educational requirements of each medical specialty^200^.

### Ethical, legal and social implications

Ethical, legal and social issues need to be considered at every step of implementation of risk-based interventions, from health-service planning, invitation of participants and consent and sample collection, to risk calculation, communication of results and storage of data^201,202^. Some of the issues associated with risk-stratified screening will be dependent on the methods by which a programme is implemented^202^.

The four principles of bio-ethics promulgated by Beauchamp and Childress^203^ — autonomy, beneficence, non-maleficence and justice — provide a useful framework to understand the potential implications of risk-based screening, although these principles are more commonly applied to the doctor–patient relationship in the clinical context. Respecting autonomy requires that an individual has adequate knowledge and understanding to decide whether they wish to opt for a given intervention. The capacity of the individual to independently make an informed decision will depend on the information content, the communication tools used and the adequacy of workforce training in conveying the relevant information. Optimizing the balance between providing benefit (beneficence) and the potential for harm (maleficence) with a risk-based screening programme requires rigorous evaluation. This balance also requires consideration and mitigation of potential unintended harms of such programmes. These unintended harms might include the negative consequences of risk assessment for individuals (such as anxiety and breaches of confidential genetic and other personal data) or at a society level (stigmatization of and discrimination against some individuals because of their risk level and non-participation of some individuals in the programme, for example, because they perceive that health care is being rationed for those for whom less screening is recommended^202^). Finally, justice relates to the fairness of a programme. Screening programmes have the potential to increase health inequalities, owing to differences in the level of uptake between socioeconomic groups, including those covered under universal health systems^204–206^. Risk-based screening programmes might exacerbate these differences^201^, given their additional complexity and inherent selectivity relative to universal screening. Efforts are needed to mitigate this possibility, for example, through ‘proportionate universalism’^207^, whereby social inequalities are considered and programme resources are targeted commensurately^208^. Communication relating to screening and risk assessment has to be accessible and congruent to the literacy and numeracy level of the recipients while also accurately presenting both the potential benefits and risks^209^. Meeting these requirements will not only avoid misinterpretation of the information provided and subsequent inequitable use of screening services, but also enable each individual to make an informed decision^201^. In addition, robust legislation is necessary to prevent discrimination and stigmatization, in particular, by insurers and employers. Current approaches vary by country, but can be broadly divided into four categories: moratoria, industry self-regulation, legal limitations to the use of genetic information, and legal bans^210,211^. As an example, in the UK, an open-ended code of practice between insurers and the government exists, prohibiting the use of predictive genetic tests except in defined circumstances^212^.

### Future directions for implementation

The time is right to perform implementation research in a real-world setting of risk-stratified prevention and screening for breast cancer, with clearly defined criteria for success (for example, relating to the extent of adoption, appropriateness, acceptability, sustainability, cost implications and effectiveness of the programme). The research should be designed and conducted together with all stakeholder groups, taking into account the ethical, legal and social context as well as factors that affect implementation (such as the idiosyncrasies of the health-care system and organizational readiness). The process has to be iterative in a health-care system conducive to learning and adaptation^213^.

To reduce the time lag between obtaining evidence on the effectiveness of a programme and its implementation, studies with hybrid effectiveness–implementation design could be used^214^ (Box 2). WISDOM^152^ and MyPeBS^153^ are examples of studies with hybrid designs primarily focused on effectiveness while also exploring the ‘implementability’ of the intervention. Several strategies adopted in WISDOM, such as the adaptive design, multistakeholder approach^215^ and CED model^174^, will accelerate implementation of the findings. By contrast, PERSPECTIVE I&I^36^ has a hybrid design focused primarily on implementation outcomes (including the acceptability and feasibility of risk-based screening, uptake of genetic testing for risk assessment and screening behaviours); however, data on effectiveness (that is, screening outcomes of different risk groups) are also being collected, and simulation modelling is being performed to assess the efficiency, resource use, costs and cost-effectiveness of risk-based screening at a population level using real-world administrative data. A third type of hybrid design involves the simultaneous study of effectiveness and implementation strategies. This approach enables the demonstration of which implementation strategies work in a given context, as opposed to demonstrating the effects of a particular implementation strategy on the adoption or uptake of an intervention^214^.

The model of evidence-generating health care could be adopted to study the clinical utility of risk stratification in the prevention of breast cancer among carriers of pathogenic *BRCA1/2* variants. This approach would require linking of genetic profiles and the outcomes of preventive interventions to cancer registries, training of treating physicians to develop a working knowledge of cancer risk and genetics, and the development of decision aids for patients.

Women with a family history of breast cancer constitute a ‘high-impact’ group in which to first pilot national level application of integrated breast cancer risk assessment. In this group, the intervention might not only substantially improve clinical management, but also provide valuable information on how risk-stratified programmes might perform in the general population. Thus, the results of this pilot approach could form the basis on which to build subsequent population-level risk-based interventions.

## Conclusions

Substantial progress has been made in research focused on estimating an individual woman’s risk of developing breast cancer, applying risk stratification in breast cancer prevention studies, modelling the benefit–harm balance of risk-stratified early detection approaches, and assessing the acceptability and feasibility of implementing risk-based prevention and screening programmes. To translate this progress into improvements in population health outcomes, a systems approach to the evaluation of risk-based programmes is necessary, taking into account the health-care organization’s readiness for change, its openness to learning and adapting, the social context and the need for engagement of all stakeholders.

## Supplementary information

Supplementary Information
