# Supplementary material for: Personalized early detection and prevention of breast cancer: ENVISION consensus statement
Source: Nat Rev Clin Oncol. 2020 Jun 18;17(11):687–705. doi: 10.1038/s41571-020-0388-9 (PMC7567644; doi:10.1038/s41571-020-0388-9)
Supplement: Supplementary file 1 — Supplementary Information [file 41571_2020_388_MOESM1_ESM.pdf]

nature REVIEWS CLINICAL ONCOLOGY

SUPPLEMENTARY INFORMATION

In format as provided by the authors

Personalized early detection and prevention of breast cancer: ENVISION consensus statement

Nora Pashayan, Antonis C. Antoniou, Urska Ivanus, Laura J. Esserman, Douglas F. Easton, David French, Gaby Sroczynski, Per Hall, Jack Cuzick, D. Gareth Evans, Jacques Simard, Montserrat Garcia-Closas, Rita Schmutzler, Odette Wegwarth, Paul Pharoah, Sowmiya Moorthie, Sandrine De Montgolfier, Camille Baron, Zdenko Herceg, Clare Turnbull, Corinne Balleyguier, Paolo Giorgi Rossi, Jelle Wesseling, David Ritchie, Marc Tischkowitz, Mireille Broeders, Dan Reisel, Andres Metspalu, Thomas Callender, Harry de Koning, Peter Devilee, Suzette Delaloge, Marjanka K. Schmidt and Martin Widschwendter

https://doi.org/10.1038/s41571-020-0388-9

Supplementary Table 1 │ **Participants of the European Conference on Risk-Stratified Prevention and Early Detection of Breast Cancer, Hall in Tirol, Austria, 26—28 June 2019**

**Delegate**	**Affiliation**
Prof. Angel Carracedo Alvarez	Center for Research in Molecular Medicine and Chronic Diseases (CiMUS), University of Santiago de Compostela, Santiago de Compostela, A Coruña, Spain.
Dr Nadine Andrieu	Institut Curie, Paris, France.
Prof. Antonis C. Antoniou	Centre for Cancer Genetic Epidemiology, Department of Public Health and Primary Care, University of Cambridge, UK.
Dr Corinne Balleyguier	Department Medical Imaging, Institut Gustave Roussy, Villejuif, France.
Dr James Barrett	Department of Women's Cancer, Institute for Women's Health, University College London, London, UK.
Dr Camille Baron	Unicancer, Paris, France.
Dr Julie Bennett	Department of Women's Cancer, Institute for Women's Health, University College London, London, UK.
Dr Proteeti Bhattacharjee	Department of Molecular Pathology, Netherlands Cancer Institute, Antoni van Leeuwenhoek Hospital, Amsterdam, The Netherlands.
Prof. Stig Bojesen	Department of Clinical Medicine, Herlev-Gentofte Hospital, University of Copenhagen, Herlev, Denmark.
Dr Ineke Bolt	Department of Medical Ethics and Philosophy of Medicine, Erasmus MC, Rotterdam, The Netherlands.
Prof. Mireille Broeders	Department for Health Evidence, Radboud University Medical Center, Nijmegen, The Netherlands.
Mr Jeroen van de Broek	Department of Public Health, Erasmus MC, Rotterdam, The Netherlands.
Dr Jean Benoit Burrion	Cancer Prevention and Screening, Medical general management, Quality unit, Institut Jules Bordet, Brussels, Belgium.
Dr Xavier Castells	Laboratory of Translational Medicine and Decision Sciences (TransLab), Department of Medical Sciences, Medicine Faculty, Universitat de Girona, Girona, Spain.
Dr Carlos Tarin Cerezo	Department of Basic Medical Sciences, Universidad CEU San Pablo, Campus de Moncloa Calle Julián Romea, Madrid, Spain.
Prof. Jenny Chang-Claude	Genetic Epidemiology Unit, Division of Cancer Epidemiology, German Cancer Research Center (DKFZ) Heidelberg, Medical Faculty, University of Heidelberg, Germany.
Prof. Georgia Chenevix-Trench	Cancer Genetics Laboratory, Department of Genetics and Computational Biology, Queensland Institute of Medical Research (QIMR) Berghofer, Brisbane, Australia.
Prof. Anna Maria Chiarelli	Dalla Lana School of Public Health, University of Toronto, Cancer Care Ontario, Toronto, Ontario, Canada.
Dr Hans Concin	AKS Vorarlberg, Austria.
Prof. Jack Cuzick	Wolfson Institute of Preventive Medicine, Barts and The London, Centre for Cancer Prevention, Queen Mary University of London, UK.
Dr Kamila Czene	Department of Medical Epidemiology and Biostatistics, Karolinska Institutet, Stockholm, Sweden.
Dr Suzette Delaloge	Breast Cancer Department, Gustave Roussy Institute, Paris, France.
Prof. Peter Devilee	Department of Human Genetics, Department of Pathology, Leiden University Medical Centre, Leiden, The Netherlands.
Dr Julia Dick	Center of Family Breast and Ovarian Cancer, University Hospital Cologne, Cologne, Germany.
Dr Alison Dunning	Centre for Cancer Genetic Epidemiology, University of Cambridge, UK.
Pro.f Douglas F. Easton	Centre for Cancer Genetic Epidemiology, Department of Public Health and Primary Care, University of Cambridge, UK.
Prof. Laurence Eloy	Centre de recherche du CHU de Québec - Université Laval, CHUL, Québec, Canada.
Dr Christoph Engel	Institute for Medical Informatics, Statistics and Epidemiology, University of Leipzig, Leipzig, Germany.
Prof. Laura Esserman	Carol Franc Buck Breast Care Center, University of California, San Francisco, San Francisco, CA, USA.
Prof. Gareth Evans	Division of Evolution & Genomic Sciences, University of Manchester, UK.
Dr Iona Evans	Department of Women's Cancer, Institute for Women's Health, University College London, London, UK.
Dr Mikael Eriksson	Department of Physiology and Pharmacology, Karolinska Institutet, Stockholm, Sweden.
Dr Angelique Flöter-Rådestad	Department of Women's and Children's Health, Karolinska Institutet, Stockholm, Sweden.
Prof. David French	Division of Psychology & Mental Health, School of Social Sciences, University of Manchester, UK.
Prof. Montserrat Garcia-Closas	Division of Cancer Epidemiology & Genetics, National Cancer Institute, Bethesda, Maryland, USA.
Dr Emilien Gauthier	Predilife, Villejuif, France.
Prof. Fiona Gilbert	Centre for Mathematical Imaging in Healthcare, University of Cambridge, UK.
Prof. Carla van Gils	Department of Epidemiology, UMC Utrecht, Division Julius Centrum, Utrecht, The Netherlands.
Dr Livia Giordano	CPO Piemonte Referral Center for Epidemiology and Oncology Prevention in Piemonte, SSD Epidemiology Screening - CRPT, Torino, Italy.
Dr Artemisa Gogollari	Institute of Public Health, Medical Decision Making and Health Technology Assessment, Department of Public Health, Health Services Research and HTA, UMIT-University for Health Sciences, Medical Informatics and Technology, Hall in Tirol, Austria.<br>Division of Health Technology Assessment, ONCOTYROL - Center for Personalized Cancer Medicine, Innsbruck, Austria.

Dr Ewan Gray	Faculty of Biology, Medicine and Health, Manchester Centre for Health Economics (MCHE), University of Manchester, UK.
Dr Michal Guindy	Assuta Medical Centers, Israel.
Dr Ivo Gut	Centro Nacional de Análisis Genómico, Centre for Genomic Regulation (CNAG-CRG), Barcelona, Spain.
Dr Kevin ten Haaf	Department of Public Health, Erasmus MC, Rotterdam, The Netherlands.
Prof. Per Hall	Department of Medical Epidemiology and Biostatistics, Karolinska Institutet, Stockholm, Sweden.<br>Department of Oncology, Södersjukhuset, Stockholm, Sweden.
Dr Lára Hallsson	Institute of Public Health, Medical Decision Making and Health Technology Assessment, Department of Public Health, Health Services Research and HTA, UMIT-University for Health Sciences, Medical Informatics and Technology, Hall in Tirol, Austria.<br>Division of Health Technology Assessment, ONCOTYROL - Center for Personalized Cancer Medicine, Innsbruck, Austria.
Dr Shaun Haran	Department of Women's Cancer, Institute for Women's Health, University College London, London, UK.
Dr Sirpa Heinävaara	Finnish Cancer Registry, Helsinki, Finland.
Ms Valerie Helin	Predilife, Villejuif, France.
Dr Zdenko Herceg	Epigenetic Group, The International Agency for Research on Cancer (IARC), WHO, Lyon, France.
Dr Weang-Kee Ho	School of Mathematical Sciences, Faculty of Science and Engineering, University of Nottingham Malaysia, Selangor, Malaysia.
Dr Miguel de la Hoya	Molecular Oncology Laboratory CIBERONC, Hospital Clínico San Carlos, IdISSC (Instituto de Investigación Sanitaria del Hospital Clínico San Carlos), Madrid, Spain.
Prof. John Hopper	Centre for Epidemiology and Biostatistics Research, Breast Cancer Unit, The University of Melbourne, Australia.
Dr Tom Hueting	Evidencio, Haaksbergen, The Netherlands.
Prof. Teo Soo Hwang	Cancer Research Malaysia, Subang Jaya Medical Centre, Subang Jaya, Selangor, Malaysia.
Dr Urška Ivanuš	Epidemiology and Cancer Registry, Institute of Oncology Ljubljana, Ljubljana, Slovenia.
Dr Beate Jahn	Institute of Public Health, Medical Decision Making and Health Technology Assessment, Department of Public Health, Health Services Research and HTA, UMIT-University for Health Sciences, Medical Informatics and Technology, Hall in Tirol, Austria.
Dr Katja Jarm	Epidemiology and Cancer Registry, DORA Registry and Call Center, Institute of Oncology, Ljubljana, Slovenia.
Ms Allison Jones	Department of Women's Cancer, Institute for Women's Health, University College London, London, UK.
Dr Susanne Knapp	Department of Women's Cancer, Institute for Women's Health, University College London, London, UK.
Ms Mona Knotek-Roggenbauer	Europa Donna - The European Breast Cancer Coalition, Austria.
Prof. Harry de Koning	Department of Public Health, Erasmus MC, Rotterdam, The Netherlands.
Dr Mateja Krajc	Ern Genturis, Institute of Oncology, Ljubljana, Slovenia.
Dr Ayse G. Kurt	Department of Obstetrics and Gynecology, Clinic of the Ludwig-Maximilians-University Munich, Germany.
Dr Anders Kvist	BioCARE: Biomarkers in Cancer Medicine improving Health Care, Education and Innovation, Lund University, Lund, Sweden.
Dr Olivia Leavy	Department of Health Sciences, Genetic Epidemiology Group Research, University of Leicester, Leicester, UK.
Dr Andreas Leimbach	Eurofins Genomics Europe Sequencing GmbH, Konstanz, Germany.
Dr Fabienne Lesueur	Institut Curie, Paris, France.
Dr Shuai Li	Centre for Epidemiology and Biostatistics Research, Breast Cancer Unit, The University of Melbourne, Australia.
Dr Jan-Willem van de Loo	European Commission, DG Research & Innovation, Unit E2 – Combatting diseases, CDMA 00/170, 1049 Brussels, Belgium.
Dr Sonia Mardinian	Unicancer, Paris, France.
Prof. Hanne Meijers-Heijboer	Department Of Clinical Genetics, Vu University Medical Center – VUMC, Amsterdam, The Netherlands.
Prof. Andres Metspalu	EGCUT - The Estonian Genome Center, University of Tartu, Estonia.
Dr Sandrine de Montgolfier	IRIS Institute for Interdisciplinary Research on Social Issues, Paris, France.
Dr Sowmiya Moorthie	PHG Foundation, Cambridge, UK.
Dr Anna Gonzalez Neira	Human Genotyping Core Unit, National Center for Oncology Research (CNIO), Madrid, Spain.
Dr Nuno Nene	Department of Women's Cancer, Institute for Women's Health, University College London, London, UK.
Dr Carolyn Nickson	Melbourne School of Population and Global Health, The University of Melbourne, Australia.
Prof. Håkan Olsson	BioCARE: Biomarkers in Cancer Medicine improving Health Care, Education and Innovation, Lund University, Lund, Sweden.
Dr Wilhelm Oberaigner	Institute of Public Health, Medical Decision Making and Health Technology Assessment, Department of Public Health, Health Services Research and HTA, UMIT-University for Health Sciences, Medical Informatics and Technology, Hall in Tirol, Austria.
Dr Tobias Paprotka	Eurofins Genomics Europe Sequencing GmbH, Konstanz, Germany.
Prof. Nora Pashayan	Department of Applied Health Research, Institute of Epidemiology and Healthcare, University College London, UK.

Prof. Paul Pharoah	Centre for Cancer Genetic Epidemiology, Department of Public Health and Primary Care, University of Cambridge, UK.
Dr Margarita Posso	Department of Epidemiology and Evaluation, IMIM (Hospital del Mar Medical Research Institute), Barcelona, Spain.
Dr Nicolien van Ravesteyn	Department of Public Health, Erasmus MC, Rotterdam, The Netherlands.
Prof. Gad Rennert	Carmel Medical Center, Technion, Clalit, Haifa, Israel.
Dr Beatriz Sobrino Rey	Universidade de Santiago de Compostela, Santiago de Compostela, Spain.
Mr David Ritchie	Faculty of Medicine and Health Sciences, University of Antwerp, Antwerp, Belgium.
Dr Daniel Roche	SOPHiA Genetics, Bidart, France.
Dr Marta Roman	Department of Epidemiology and Evaluation, IMIM (Hospital del Mar Medical Research Institute), Barcelona, Spain.
Dr Atocha Romero	Department of Medical Oncology, Puerta de Hierro-Majadahonda University Hospital, Madrid, Spain.
Dr Matti Rookus	Division Psychosocial Research and Epidemiology, Netherlands Cancer Institute, Antoni van Leeuwenhoek Hospital, Amsterdam, The Netherlands.
Dr Paolo Giorgi Rossi	Epidemiology Service, Azienda USL of Reggio Emilia, IRCCS, Reggio Emilia, Italy.
Dr Tytti Sarkeala	Finnish Cancer Registry, Helsinki, Finland.
Dr Marjanka Schmidt	Department of Molecular Pathology, Netherlands Cancer Institute, Antoni van Leeuwenhoek Hospital, Amsterdam, The Netherlands.
Prof. Rita Schmutzler	Center of Family Breast and Ovarian Cancer, University Hospital Cologne, Cologne, Germany.
Prof. Nereo Segnan	Department of Cancer Screening and Unit of Cancer Epidemiology, Center for Epidemiology and Prevention in Oncology, CPO Piedmonte, WHO Collaborative Center for Cancer Early Diagnosis and Screening, Torino, Italy.
Prof. Uwe Siebert	Institute of Public Health, Medical Decision Making and Health Technology Assessment, Department of Public Health, Health Services Research and HTA, UMIT-University for Health Sciences, Medical Informatics and Technology, Hall in Tirol, Austria.<br>Division of Health Technology Assessment, ONCOTYROL - Center for Personalized Cancer Medicine, Innsbruck, Austria.
Prof. Sabine Siesling	Netherlands Comprehensive Cancer Organisation (IKNL), Department Research and Development, Utrecht, The Netherlands.<br>University of Twente, Technical Medical Centre,<br>Department Health Technology and Services Research, Enschede, The Netherlands.
Prof. Jacques Simard	CHU de Québec - Université Laval Research Center, Genomics Center, Québec, Canada.
Dr Efrat Slonim	Assuta Medical Centers, Israel.
Prof. Melissa Southey	Genetic Epidemiology Laboratory (GEL), Department of Pathology, The University of Melbourne, Australia.
Dr Gaby Sroczynski	Institute of Public Health, Medical Decision Making and Health Technology Assessment, Department of Public Health, Health Services Research and HTA, UMIT-University for Health Sciences, Medical Informatics and Technology, Hall in Tirol, Austria.<br>Division of Health Technology Assessment, ONCOTYROL - Center for Personalized Cancer Medicine, Innsbruck, Austria.
Prof. Ewout Steyerberg	Department of Public Health, Erasmus MC, Rotterdam, The Netherlands.
Prof. Dominique Stoppa-Lyonnet	Institut Curie, Paris, France.
Dr Karin Sundström	Department of Laboratory Medicine, Karolinska Institutet, Stockholm, Sweden.
Dr Francois Taban	Clinique Générale-Beaulieu, Geneva, Switzerland.
Dr Marc Tischkowitz	Department of Medical Genetics, National Institute for Health Research Cambridge Biomedical Research Centre, University of Cambridge, Cambridge, UK.
Dr Krista Tromp	Department of Medical Ethics and Philosophy of Medicine, Erasmus MC, Rotterdam, The Netherlands.
Prof. Clare Turnbull	Division of Genetics and Epidemiology, Institute Of Cancer Research, London, UK.
Dr Lucie Veron	Institut Gustave Roussy, Villejuif, France.
Dr Benjamin Verret	Institut Gustave Roussy, Villejuif, France.
Dr Cecile Vissac-Sabatier	French Breast Cancer Intergroup, Unicancer, Paris, France.
Dr Odette Wegwarth	Max Planck Institute for Human Development, Center for Adaptive Rationality, Harding Center for Risk Literacy, Berlin, Germany.
Prof. Jelle Wesseling	Division of Molecular Pathology, Netherlands Cancer Institute, Antoni van Leeuwenhoek Hospital, Amsterdam, The Netherlands.
Prof. Martin Widschwendter	Department of Women's Cancer, Institute for Women's Health, University College London, London, UK.
Mrs Jiran Vatankhah Atashgah	Department of Women's Cancer, Institute for Women's Health, University College London, London, UK.
Dr Maaike Vreeswijk	Leiden University Medical Center, Leiden, The Netherlands.
Dr Maryam Yahiaoui-Doktor	Institute for Medical Informatics, Statistics and Epidemiology, University of Leipzig, Germany.
